# Oedema reduction mediates thrombectomy benefit in large core stroke: secondary analysis of the TENSION trial

**DOI:** 10.1093/esj/aakag055

**Published:** 2026-06-04

**Authors:** Gabriel Broocks, Martin Bendszus, Claus Z Simonsen, Götz Thomalla, Elke R Gizewski, Anne Hege Aamodt, Antonin Krajina, Laurent Pierot, Kamil Zeleňák, Blanca Fuentes, Michael D Hill, Andre Kemmling, Lieselotte Vandewalle, Anke Wouters, Robin Lemmens, Jelle Demeestere, Susanne Gellißen, Jens Fiehler, Lukas Meyer, Helge Kniep

**Affiliations:** Department of Diagnostic and Interventional Neuroradiology, University Medical Center Hamburg-Eppendorf, Hamburg, Germany; Department of Neuroradiology, HELIOS Medical Center, University Campus of MSH Medical School Hamburg, Schwerin, Germany; Universitätsklinikum Heidelberg, National Coordinating Center, Heidelberg, Germany; Aarhus University Hospital, National Coordinating Center, Aarhus, Denmark; Department of Neurology, University Medical Center Hamburg-Eppendorf, Hamburg, Germany; Department of Radiology, Medical University Innsbruck, National Coordinating Center, Innsbruck, Austria; Oslo University Hospital, National Coordinating Center, Oslo, Norway; Charles University, Hradec Králové, National Coordinating Center, Hradec Králové, Czech Republic; Centre Hospitalier Universitaire de Reims, National Coordinating Center, Reims, France; Jessenius Faculty of Medicine, Comenius University and University Hospital Martin, National Coordinating Center, Martin, Slovakia; La Paz University Hospital, National Coordinating Center, Madrid, Spain; University of Calgary, National Coordinating Center, Calgary, AB, Canada; Department of Neuroradiology, University of Marburg, Marburg, Germany; Division of Experimental Neurology, Department of Neurosciences, KU Leuven, Belgium; Department of Neurology, Leuven University Hospital, Leuven, Belgium; Division of Experimental Neurology, Department of Neurosciences, KU Leuven, Belgium; Division of Experimental Neurology, Department of Neurosciences, KU Leuven, Belgium; Department of Neurology, Leuven University Hospital, Leuven, Belgium; Division of Experimental Neurology, Department of Neurosciences, KU Leuven, Belgium; Department of Neurology, Leuven University Hospital, Leuven, Belgium; Department of Diagnostic and Interventional Neuroradiology, University Medical Center Hamburg-Eppendorf, Hamburg, Germany; Department of Diagnostic and Interventional Neuroradiology, University Medical Center Hamburg-Eppendorf, Hamburg, Germany; Department of Diagnostic and Interventional Neuroradiology, University Medical Center Hamburg-Eppendorf, Hamburg, Germany; Department of Diagnostic and Interventional Neuroradiology, University Medical Center Hamburg-Eppendorf, Hamburg, Germany

**Keywords:** stroke, imaging, thrombectomy, oedema

## Abstract

**Purpose:**

Several recent trials have shown improved functional outcomes with endovascular thrombectomy (EVT) in patients presenting with large ischaemic lesions, yet the impact of reperfusion on lesion pathophysiology in this population remains incompletely understood. We hypothesised that EVT reduces ischaemic oedema formation and that attenuation of oedema mediates functional outcome improvement beyond changes in lesion extent.

**Methods:**

We performed a secondary analysis of the randomised TENSION trial, which enrolled patients with anterior circulation ischaemic stroke and baseline ASPECTS 3–5. Oedema progression was quantified as the change in CT-based net water uptake between baseline and follow-up non-contrast CT (ΔNWU), derived from relative hypoattenuation within the infarct. Lesion extent change was assessed by the difference in ASPECTS between baseline and follow-up imaging (ΔASPECTS). Associations of ΔNWU and ΔASPECTS with 90-day modified Rankin Scale (mRS) were tested using ordinal logistic regression. Mediation analyses estimated the proportion of EVT’s treatment effect on functional outcome explained by ΔNWU and ΔASPECTS.

**Results:**

Among 177 patients, median follow-up NWU was 28.5% (IQR: 19.2–36.6) and follow-up ASPECTS was 2 (IQR: 1–3). EVT was associated with substantially lower oedema progression (ΔNWU 4.7% vs 15.9% without EVT; *P* < .001). Mediation analysis suggested that a model-estimated 34% of EVT-related improvement in mRS was associated with reduced oedema formation (ΔNWU), whereas 14% was associated with lesion extent change (ΔASPECTS). In a sensitivity analysis using nonparametric bootstrap with 5000 resamples, the mediation signal remained robust for ΔNWU but was substantially weaker for ΔASPECTS.

**Conclusion:**

In large-core stroke, EVT was associated with significantly attenuated oedema progression between baseline and follow-up imaging. Reduced ischaemic water uptake was strongly associated with EVT benefit on 90-day functional outcome in mediation models, supporting oedema attenuation as a plausible mechanistic pathway of EVT benefit.

## Graphical Abstract

**Graphical Abstract:**
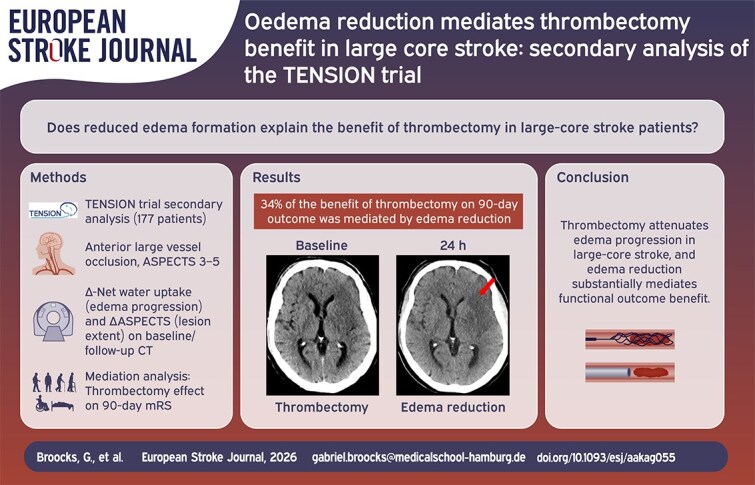

## Introduction

Recent randomised controlled trials have demonstrated that endovascular thrombectomy (EVT) substantially improves functional outcomes and survival in patients with anterior circulation large-vessel occlusion strokes compared to best medical treatment (BMT) alone, even in subgroups presenting with large ischaemic lesions visible on standard admission CT imaging.^1–4^ Despite these advances, the impact of EVT on lesion pathophysiology in patients with large infarctions and limited tissue at risk is generally not yet well understood, particularly considering that even patients with ASPECTS 0–2 benefitted from EVT.^2^^,^^5^ Hence, secondary treatment effects of recanalisation may play a pivotal role explaining the treatment effect in large strokes, as previously observed in several retrospective studies.^5–11^ The evidence of a treatment effect independent of penumbra salvage and baseline lesion extent would have important clinical implications regarding treatment decision-making in acute LVO stroke. Lesion hypoattenuation on non-contrast CT is traditionally equated with irreversible infarction because it reflects tissue water accumulation.^12–14^ Experimental and clinical evidence, however, indicate that early hypoattenuation can still be reversible when the blood–brain barrier (BBB) remains intact and the water shift is driven mainly by sodium-ion disequilibrium. This ionic oedema reflects the earliest phase of endothelial dysfunction.^15^ Successful reperfusion during this phase may not only salvage penumbral tissue but might also arrest or even reverse hypoattenuation/oedema.^16^ Visible hypoattenuation on non-contrast CT has long been interpreted as the imaging correlate of infarct core and therefore as tissue beyond salvage. However, that paradigm may be incomplete: if early CT hypoattenuation partly reflects ionic oedema before BBB disruption, some apparently infarcted tissue may still remain reversible with timely reperfusion.^17^^,^^18^ Observational data further suggest that a small proportion of patients undergoing revascularisation may show true reversal of tissue hypoattenuation on follow-up imaging and that reduction in net water uptake (NWU) may capture a related form of oedema reversibility. Together, these observations raise the possibility that some early CT hypoattenuation represents potentially reversible injury and that reperfusion may beneficially modify subsequent oedema formation.^7^^,^^19^^,^^20^

The purpose of this study was to evaluate the extent to which the observed EVT-related improvement in 90-day functional outcomes in large strokes is mediated by (1) oedema reduction, quantified by NWU and (2) reduction in lesion-extent progression, quantified by the difference in ASPECTS between baseline and follow-up imaging (ΔASPECTS), as assessed on baseline and follow-up imaging. We hypothesised 2-fold that (1) patients undergoing EVT show less oedema progression compared to those receiving BMT alone and (2) oedema reduction significantly mediates the effect of EVT on functional outcome.

## Methods

### Study design

TENSION (The Efficacy and Safety of Thrombectomy in Stroke with extended lesion and extended time window, NCT03094715) was an investigator-initiated, randomised, open-label, blinded endpoint, 2-arm, post-market trial in Europe and Canada. The study design and inclusion criteria have been described before.^1^ Further details can be found in the Supplementary material. The study protocol was approved by the Ethics Committee of the Medical Faculty of Heidelberg University (S-658/2017) and the institutional review boards of all participating centres. Informed consent was obtained from all participants or their legal representatives.

### Study cohort

For the present secondary analysis, we included only trial participants with baseline non-contrast CT (NCCT) and 24-h follow-up NCCT suitable for quantitative oedema assessment and lesion progression analysis. Patients were excluded if the CT imaging data required for this analysis were not available or not suitable for quantitative CT-based assessment of NWU and ASPECTS. Although the parent TENSION trial allowed either CT-based imaging (NCCT/CTA) or MRI-based imaging (DWI/MRA) at baseline according to local institutional preference, patients imaged primarily with MRI were not eligible for this CT-based substudy.

### Clinical assessment

All patients underwent clinical assessments on admission, 24 h (±6) post-treatment, at 7 days or upon hospital discharge and at 90 days (±14). The 90-day clinical assessment was conducted either in-person or via telephone interview by trained personnel who were blinded to the treatment group assignments.

### Outcome measures

Primary outcome was the ordinal distribution of functional neurological disability scored on the modified Rankin Scale (mRS) at 90 (±14) days, analysed using an ordinal (shift) model with all 7 categories retained (0–6; mRS 5 and 6 not merged). The degree of recanalisation was evaluated using the modified thrombolysis in cerebral infarction (mTICI) scale. Successful recanalisation was defined as mTICI 2b-3, and complete recanalisation was defined as mTICI 2c-3. sICH was defined as the presence of any intracranial haemorrhage on follow-up imaging within 24 h and a neurological worsening of ≥4 points on the NIHSS^21^ in accordance with ECASS. Malignant oedema (MMI) on follow-up NCCT was defined according to the trial imaging protocol as a large space-occupying infarct with visually evident mass effect, including marked sulcal and ventricular effacement and the presence of midline shift. Effacement was assessed visually and was not graded on a separate ordinal scale; likewise, midline shift was not systematically quantified in millimetres but evaluated as part of the overall imaging impression of space-occupying infarction. MMI was recorded as an exploratory trial-based imaging variable and not intended as the primary quantitative marker of oedema severity in the present analysis.

### Image analysis

Baseline and follow-up CT imaging data were processed and analysed by an independent imaging core laboratory (Eppdata, Hamburg, Germany). All patients underwent a standardised protocol with non-contrast CT (NCCT) and CTA; CTP was not required for enrolment in TENSION. NWU was assessed using an established image processing pipeline.^22^ NCCT and CTA datasets were rigidly co-registered to ensure spatial alignment. Co-registered images were imported into ITK-SNAP (Version 4.0.1). CTA source images (CTA-SI) were used to guide the initial segmentation of the region of interest (ROI), serving as a surrogate for CBV maps by indicating areas with reduced intravascular and parenchymal contrast enhancement consistent with impaired microvascular filling. A mirrored contralateral ROI was generated automatically. Cerebrospinal fluid and sulci were excluded by intensity-based thresholding. Final ROI validation and all quantitative measurements were performed on NCCT.^22^ The same approach was used on follow-up NCCT, with particular attention to possible space-occupying infarction; in such cases, the contralateral ROI was manually adjusted to account for mass effect. For baseline and follow-up imaging, the density distribution (in Hounsfield Units, HU) was measured within the ischaemic lesion and the mirrored contralateral normal tissue. Median densities D*_ischaemic_* and D*_normal_* were derived from histograms within the range of 20–80 HU, as previously validated.^12^^,^^23^ NWU on NCCT was calculated according to the following formula, as previously reported^12^^,^^23^:

NWU (%) = (1 - D*_ischaemic_*/D*_normal_*)×100

All segmentations and measurements for NWU calculations were performed by experienced, board-certified neuroradiologists, blinded to all clinical data. The regional extent of the ischaemic lesion was quantified based on ASPECTS ratings of both baseline and follow-up NCCT, and lesion-extent change was defined as ΔASPECTS (follow-up minus baseline). ASPECTS was selected as the primary metric of infarct extent because validated, Core Lab–assessed ASPECTS ratings were consistently available at both time points across the entire cohort, thereby enabling trajectory analyses directly comparable to net water uptake between baseline and follow-up non-contrast CT (ΔNWU).

### Statistical analysis

Statistical analyses were performed on the modified as-treated cohort, adhering to the study’s predefined inclusion and exclusion criteria. Continuous data were reported as medians with IQR, while categorical variables were expressed as absolute counts and percentages. Group comparisons for categorical data were assessed using the χ^2^ test. To assess the normality of continuous data, the Kolmogorov–Smirnov test was applied. For continuous variables with normal distribution, comparisons were made using Student’s *t*-tests along with CI. For non-normally distributed continuous data, the Mann–Whitney U test was used, with results reported as IQR. Baseline, procedural and outcome variables were compared between patients randomised to BMT and those receiving EVT.

To evaluate the relationship between baseline and treatment variables and functional outcome, multivariable ordinal logistic regression analysis was conducted with the mRS at day 90 as the dependent variable. Odds ratios (OR) with corresponding 95% CI and *P*-values were reported. For the ordinal models, the proportional-odds assumption was formally tested. In addition, linear regression analysis with backward variable selection was used to identify independent predictors of ΔNWU and ΔASPECTS. Because ASPECTS and NWU describe different characteristics of the same ischaemic lesion, we examined potential multicollinearity between them. ASPECTS reflects lesion extent within the affected vascular territory, whereas NWU quantifies the degree of ischaemic water uptake within the lesion and therefore captures oedema severity independently of lesion size. We assessed their relationship by reporting correlations between baseline, follow-up and ΔASPECTS and the corresponding NWU measures and by calculating variance inflation factors (VIFs) from centred and scaled models. Time from admission to follow-up CT (tFU) was included as a covariate in all models involving ΔNWU. In addition, ΔNWU was compared between patients with earlier versus later follow-up imaging (tFU ≤ 24 h vs > 24 h).

Mediation analyses were performed to evaluate the extent to which oedema reduction and changes in lesion extent following EVT contribute to functional outcomes at 90 days (see Supplementary material). Mediation models were adjusted for prespecified baseline covariates associated with stroke severity, tissue injury and outcome, including age, baseline NIHSS, baseline ASPECTS and time from symptom onset to imaging; in analyses involving ΔNWU, time from admission to follow-up CT was additionally included. We did not adjust for post-randomisation factors such as ICU-based measures, decompressive surgery or blood pressure management, because these variables were not available in a sufficiently standardised manner across the cohort and may themselves be influenced by treatment allocation and oedema progression. As a sensitivity analysis, indirect effects were additionally re-estimated using nonparametric bootstrap with 5000 resamples, and bootstrap confidence intervals were examined to assess the robustness of the mediation findings. Because the mediators were measured on follow-up imaging after treatment, mediation effects were interpreted as model-based estimates under standard mediation assumptions rather than as definitive causal decompositions. A 2-tailed *P* < .05 was considered significant for all statistical tests. No adjustments were made for multiple comparisons. Analyses were performed using StataNow/MP 18.5 (Stata Corp, TX, USA). The study was reported using the Consolidated Standards of Reporting Trials (CONSORT) reporting guideline.

## Results

### Study cohort baseline characteristics

**Figure 1:**
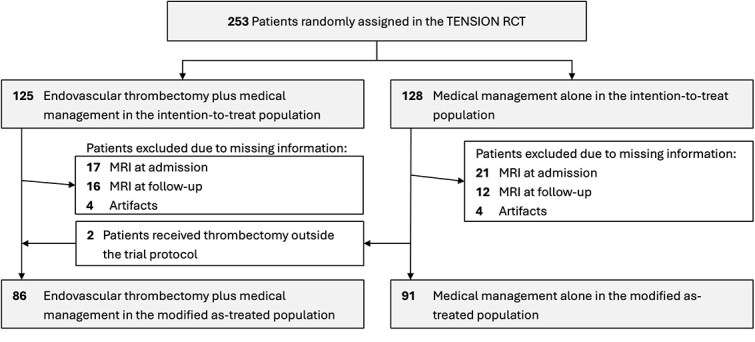
Flow chart of patient inclusion. Of 253 TENSION trial patients, 177 had both baseline and follow-up non-contrast CT (NCCT) and comprised the analysed cohort; allocation to endovascular thrombectomy (EVT) + best medical treatment (BMT) vs BMT-only and reasons for exclusion (eg, missing follow-up NCCT) are shown. Among EVT-treated patients, successful reperfusion mTICI2b/3 occurred in 87%.

**Figure 2:**
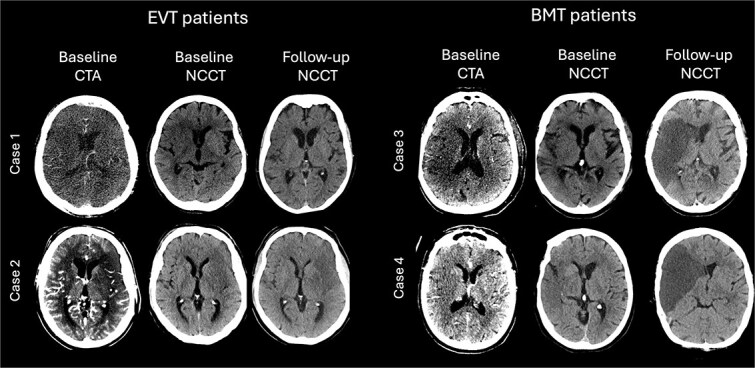
Patient examples. Four patients (case 1/2: EVT, case 3/4: BMT) showing baseline imaging and follow-up CT. In accordance with the main results of this study, follow-up imaging shows a significant reduction in oedema formation after EVT. Case 1/2: ΔNWU of 2.0%/1.5%; case 3/4: ΔNWU: 20.4%/19.6%.

After screening of the 253 patients included in the trial, 177 were included in this secondary analysis, whereas 76 were not included. A flow chart of patient inclusion is shown in Figure 1. A case illustration is shown in Figure 2. Patients were excluded primarily for methodological reasons, because the CT imaging data required for quantitative assessment of NWU and ASPECTS were either not available or not suitable for analysis. This mainly concerned patients imaged primarily with MRI (*n* = 41) and patients with CT-based imaging that was not usable for the present measurements or lacked evaluable follow-up CT (*n* = 35). Included and non-included patients did not show major baseline differences: median age was 74 versus 72 years, median NIHSS 18 versus 19 and median baseline ASPECTS 4 in both groups. In the analysed CT cohort, the difference between as-treated and intention-to-treat grouping was minimal, as treatment deviations were rare (as-treated: 91 BMT vs 86 EVT; ITT differed by only 2 patients).

The median age was 74 (IQR: 65–80), and the median NIHSS was 18 (IQR: 15–22). The median ASPECTS was 4 (IQR: 3–5). On follow-up CT, the median ASPECTS was 2 (IQR: 1–3). In 40 patients (23%), the ASPECTS did not decrease (ie, no increased lesion extent) from baseline to follow-up. In 137 patients (77%) ASPECTS declined (−1 = 36 patients; −2 = 32 patients; −3 = 39 patients; −4/5 = 30 patients). The median NWU at baseline was 16.4% (IQR: 11.7−20.3) and the median NWU at follow-up was 28.5% (IQR: 19.2−36.6). Seventy-three patients (41%) had received intravenous treatment with alteplase. In 85 patients (48%), EVT was performed, of which 74 patients (87%) achieved successful recanalisation (mTICI 2b-3). At day 90, the median mRS was 5 (IQR: 4–6). The rate of independent ambulation (mRS 0–3) was 20% (35 patients). The rate of sICH was 5.3% (9 patients) (Table 1A).

**Table 1:** Study cohort baseline characteristics (A) and study cohort primary and secondary outcomes, procedural success and safety events (B).

A	All patients	BMT	MT	*P*
**Age in years,** **median (IQR)**	74.0 (65.0–81.0)	74.0 (65.5–81.0)	73.5 (64.2–80.5)	.67
**Baseline NIHSS,** **median (IQR)**	18.0 (15.0–22.0)	18.0 (15.0–21.0)	18.0 (16.0–22.0)	.38
**Baseline ASPECTS,** **median (IQR)**	4.0 (3.0–5.0)	4.0 (3.0–5.0)	4.0 (3.2–5.0)	.19
**Time onset—randomisation, h** **median (IQR)**	4.8 (3.4–6.9)	4.6 (3.4–6.5)	4.2 (3.2–6.2)	.67
**Sex female, n**	84 (47.5%)	48 (52.7%)	36 (41.9%)	.19
**Intravenous thrombolysis**	72 (40.7%)	36 (39.6%)	36 (41.9%)	.87
**B**	**All patients**	**BMT**	**MT**	***P***
**Baseline NWU**	16.1 (11.6–20.4)	17.5 (13.0–21.3)	14.9 (11.2–18.1)	.02
**Follow-up NWU**	28.6 (19.3–36.6)	34.0 (28.4–39.8)	19.8 (13.5–29.1)	<.01
**ΔNWU, %-points**	11.8 (3.0–19.1)	15.9 (9.7–21.7)	4.8 (−1.1 to 12.3)	<.01
**ΔASPECTS**	2 (1–3)	2.0 (1.0–3.0)	2.0 (0.0–3.0)	.20
**NIHSS at 24 h**	18 (14–22)	19.0 (16.0–23.0)	15.0 (8.5–20.0)	<.01
**ΔNIHSS (BL → 24 h)**	0 (−2 to 4)	−1.0 (−3.0 to 1.0)	4.0 (−0.5 to 7.5)	<.01
**mRS day 90,** **median (IQR)**	5.0 (4.0–6.0)	6.0 (5.0–6.0)	5.0 (3.0–6.0)	<.01
**Malignant infarction on follow-up, n(%)**	37 (21.3%)	19 (21.1%)	18 (21.4%)	1.00

Abbreviations: BL = baseline; EVT = endovascular thrombectomy; IVT = intravenous thrombolysis; LSW = last seen well; mRS = modified Rankin Scale; NWU = net water uptake. “All” column represents full as-treated cohort (*n* = 177).

At follow-up, NWU was significantly higher (34% versus 19%, *P* < .001) and ASPECTS significantly lower (2 versus 3, *P* = .02) comparing patients in the BMT versus EVT group. ∆NWU and ∆ASPECTS were different comparing BMT to EVT patients: the median ∆NWU was 16% versus 5% (*P* < .001) and ∆ASPECTS was 2 in both group (*P* = .05). The mean time from admission to follow-up CT (tFU) was 25 h, with a median of 24 h (5th percentile 16 h). ΔNWU did not differ significantly between patients with earlier versus later follow-up imaging (median ΔNWU 0.10 vs 0.12, Wilcoxon rank-sum z = −1.60, *P* = .11), and adjustment for tFU did not change the association between EVT, ΔNWU and functional outcome. This pattern was similar after stratification by treatment: in the BMT group, median ΔNWU was 0.15 vs 0.17 for earlier versus later follow-up imaging (Wilcoxon rank-sum z = −1.50, *P* = .14), and in the EVT group, median ΔNWU was 0.02 vs 0.09 (z = −1.75, *P* = .08) (Table 1B).

Correlations between ASPECTS and NWU measures were modest: across baseline, follow-up and change variables. Pearson correlation coefficients ranged from −0.35 to 0.27, with the strongest correlation observed between follow-up ASPECTS and follow-up NWU (*r* = −0.35). In the linear model including treatment group (BMT vs EVT), ΔNWU, ΔASPECTS, age and baseline NIHSS, all variance inflation factors were low (range 1.05–1.35; VIF for ΔNWU = 1.35, VIF for ΔASPECTS = 1.05), indicating no relevant multicollinearity.

### Mediation pathways—association of treatment and outcome with ∆NWU and ΔASPECTS

#### Association of EVT and functional outcome

In univariable ordinal regression analysis, EVT was significantly associated with a lower mRS score at day 90 (cOR: 0.39, 95% CI, 0.25–0.64, *P* < .001). This association was confirmed in multivariable analysis including age, NIHSS, ASPECTS and time from onset to imaging (acOR: 0.34, 95% CI, 0.21–0.55, *P* < .001) (Table 2).

**Table 2:** Univariable and multivariable ordinal regression analysis for primary outcome modified Rankin Scale score (mRS) at day 90.

	Univariable analysis, outcome mRS at 90 days			Multivariable analysis, outcome mRS at 90 days		
	OR	95% CI	*P*-value	acOR	95% CI	*P*-value
**Primary outcome mRS at 90 days**						
**ΔNWU**	1.06	1.03–1.10	<.01	1.04	1.01–1.08	.01
**EVT**	0.30	0.17–0.54	<.01	0.39	0.19–0.79	<.01
**ΔASPECTS**	1.55	1.28–1.87	<.01	1.66	1.35–2.05	<.01

Multivariable ordinal regression models were adjusted for: age, sex, baseline pre-stroke mRS, NIHSS, ASPECTS, use of intravenous alteplase and EVT. ΔNWU expressed per 1 %-point increase; ΔASPECTS per 1-point increase (greater decline). Abbreviations: acOR = adjusted common odds ratio; OR = odds ratio.

#### Association of EVT and ∆NWU/ΔASPECTS

In univariable linear regression analysis, EVT was significantly associated with lower oedema formation from baseline to follow-up CT (∆NWU, ß: −0.10, 95%, CI, −0.13 to −0.71, *P* < .001). Also, EVT was associated with a lower ΔASPECTS (ß: −0.50, 95% CI, −0.9 to −0.03, *P* = .04). In multivariable linear regression analysis, the independent association of ∆NWU (aß: −0.1, 95% CI, −0.13 to −0.07, *P* < .001) and ΔASPECTS (aß: −0.49, 95% CI, −0.95 to −0.04, *P* = .03) with EVT was confirmed.

#### Association of ∆NWU/ASPECTS difference and functional outcome

In univariable ordinal regression analysis, ∆NWU was significantly associated with functional outcome at day 90 (cOR: 1.06, 95% CI, 1.03–1.09, *P* < .001). Similarly, ΔASPECTS was associated with functional outcome (cOR: 1.48, 95% CI, 1.27–1.72, *P* < .001). Multivariable analysis confirmed the independent association for both imaging items with functional outcome (∆NWU, acOR: 1.07, 95% CI, 1.04–1.11, *P* < .001), ΔASPECTS (acOR: 1.56, 95% CI, 1.33–1.83, *P* < .001) (Table 2).

### Mediation analysis

Mediation models are illustrated in Figure S1. EVT was associated with a 10.05-point lower ΔNWU (path a, *P* < .001), and larger ΔNWU was independently linked to worse mRS at day 90 (path b, *P* < .001). The total EVT effect on 90-day mRS was −0.93 mRS points, and the indirect effect through ΔNWU was −0.32 mRS points. Within this mediation framework, the model-estimated proportion of the overall EVT effect associated with oedema reduction was 34%. By contrast, the corresponding model-estimated proportion associated with ΔASPECTS was 14%. These estimates were used to compare the relative strength of the 2 imaging pathways and should not be interpreted as precise causal decompositions. As a sensitivity analysis, mediation effects were re-estimated using nonparametric bootstrap with 5000 resamples. This analysis yielded directionally similar results and confirmed a robust indirect effect of EVT through ΔNWU, whereas the mediation signal for ΔASPECTS remained smaller and less robust. Detailed bootstrap estimates are provided in the Supplement.

In an exploratory analysis with MMI as the outcome and ΔNWU as mediator, EVT was associated with lower ΔNWU (path a, β = −0.10, *P* < .001), and higher ΔNWU was associated with a higher probability of malignant oedema (path b, β = +0.94, *P* = .003). The indirect effect of EVT on MMI through ΔNWU was significant (β_indirect = −0.09; 95% CI −0.17 to −0.03; *P* ≈ .007), whereas the direct effect of EVT on MMI after accounting for ΔNWU was small and non-significant (β_direct = +0.10, *P* = .16). This pattern is compatible with indirect-only mediation, suggesting that EVT reduces malignant oedema predominantly via attenuation of oedema formation as quantified by ΔNWU.

## Discussion

This study investigated the role of oedema reduction as a mediator of the beneficial effects of EVT in patients with large infarct cores, using quantitative NWU as an imaging biomarker. Our findings support the hypothesis that, in addition to effects on infarct volume and perfusion, EVT exerts a significant effect on the pathophysiology of brain tissue injury by limiting cytotoxic and vasogenic oedema formation.

Notably, mediation models suggested that a model-estimated 34% of the overall treatment effect of EVT on functional outcomes at 90 days was associated with oedema reduction, whereas the corresponding estimate for lesion-extent change was 14%. These values should be interpreted as approximate, model-dependent indicators within the applied mediation framework rather than as precise causal quantities. Nevertheless, the comparison supports the view that oedema dynamics may represent an important secondary pathophysiological pathway of EVT benefit. Sensitivity analyses using nonparametric bootstrap with 5000 resamples yielded directionally similar results, confirming a robust signal for ΔNWU, whereas the corresponding mediation signal for ΔASPECTS remained substantially weaker. Given the post-treatment measurement of the mediator and the potential influence of subsequent clinical factors, these estimates should be interpreted as mechanistically informative rather than as proof of a fully confounder-free causal pathway.

It is important to emphasise that ASPECTS and NWU capture complementary dimensions of ischaemic injury. ASPECTS primarily reflects lesion extent, whereas NWU quantifies the degree of tissue hypoattenuation and thus the “depth” of ischaemia and oedema within the affected territory.^14^^,^^24^ Ischaemic lesions with identical ASPECTS can therefore exhibit markedly different NWU values, indicating different water content and, likely, different risks of malignant swelling and poor outcome. This conceptual distinction explains why both ΔASPECTS and ΔNWU were independently associated with functional outcome in our models, and why oedema reduction (ΔNWU) mediated a larger proportion of EVT benefit than changes in lesion extent.^14^^,^^23^

From a pathophysiological perspective, cerebral oedema evolves in 2 overlapping phases. Ionic oedema—characterised by preserved BBB integrity but dysfunctional ion homeostasis—dominates the first hours, whereas vasogenic oedema follows BBB breakdown.^15^ Accumulation of extracellular fluid, and subsequent mass effect worsen neurological function and contribute to poor outcomes. In large infarcts, where viable tissue for penumbral salvage is limited, these secondary injury processes can substantially influence prognosis.^10^^,^^11^^,^^25^ By re-establishing flow during the ionic oedema window, EVT may halt the sodium-driven fluid shift and thus curb subsequent vasogenic swelling. At the same time, EVT benefit in large-core stroke is likely multifactorial and may not be explained by oedema attenuation alone. Other plausible contributors include selective regional vulnerability within the ischaemic lesion and differential susceptibility of grey and white matter to ischaemia and reperfusion.

Our findings demonstrate that EVT reduces the degree of NWU, suggesting that EVT can limit the severity of oedema formation beyond its effect on final infarct size. These observations support the concept that improved blood flow may mitigate subsequent oedema formation, even in tissue with advanced ischaemic injury. These results align with previous experimental and clinical studies suggesting that rapid reperfusion can reduce the permeability of the BBB.^26–30^

In this study, the observed mediation effect of oedema reduction on functional outcome highlights the importance of this mechanism in the context of large ischaemic cores, where direct salvage of penumbral tissue is comparably limited. The observation that the model-estimated mediation signal for oedema reduction was larger than that for lesion-extent change supports the relevance of oedema dynamics for functional outcomes in patients with low ASPECTS^8^ and complements smaller reports linking early NWU with malignant infarction and decompressive craniectomy.^23^^,^^31–33^

Our findings extend prior work in large-core and low-ASPECTS populations, where several observational and trial-based analyses have reported heterogeneous results regarding oedema dynamics, final infarct volume and functional outcome.^8^^,^^34^^,^^35^ Some studies have emphasised the role of oedema and NWU as key mediators of treatment effect, whereas others, including analyses using midline shift as the primary oedema metric, have yielded conflicting conclusions.^26^^,^^30^^,^^36^ By leveraging randomised TENSION data and explicitly modelling ΔNWU as a mediator between EVT and outcome, our study adds complementary evidence focused on oedema evolution rather than solely on final infarct volume.^5^^,^^25^^,^^34^

A retrospective multicentre cohort of low-ASPECTS strokes reported a larger oedema-related mediation estimate (>60%).^11^ Differences from our findings may reflect residual confounding in non-randomised data, differences in covariate adjustment and differences in treatment definitions, as that study compared patients with and without angiographic reperfusion rather than EVT versus BMT. At the same time, our own mediation estimates should also be interpreted cautiously, as they remain model-dependent and rely on assumptions that cannot be fully verified in a post-treatment mediator setting.

Alternative oedema metrics such as midline shift (MLS) were not systematically quantified by the TENSION imaging core laboratory and were therefore unavailable for robust analysis in the present cohort. Conceptually, MLS is a late and indirect surrogate of oedema that depends heavily on pre-existing intracranial reserve; older patients with substantial atrophy may accumulate marked oedema without developing significant MLS, whereas younger patients with tight intracranial compartments may exhibit pronounced MLS despite more modest water uptake.^24^^,^^37^ In contrast, NWU directly quantifies tissue water content within the infarct and is applicable across the full range of lesion sizes, including those without visible MLS. A recent secondary analysis of ANGEL-ASPECT by Nie et al. defined MLS at 24 (±12) hours as the primary oedema outcome and reported that EVT was associated with a numerically small increase in MLS compared with medical management (mean 3.0 vs 2.4 mm; difference 0.6 mm). Although this group-level difference reached statistical significance, it is far below the thresholds usually considered clinically meaningful for mass effect and well within the range of typical measurement variability for manual MLS assessment, making its biological and clinical significance uncertain. Notably, in the same work the progression of NWU remained slower after EVT throughout 7 days, which is entirely consistent with an oedema-limiting effect of reperfusion despite the small MLS signal. These findings underscore that MLS and NWU capture different aspects of the pathophysiology (downstream mass effect vs. early water content).^38^ In contrast, multiple independent studies have linked CT-based NWU and NWU-derived oedema volume to infarct age, malignant oedema and poor functional outcome, and have shown that NWU modifies the treatment effect of EVT in large-core populations.^6^^,^^22^^,^^39^ Our additional mediation analysis with malignant oedema as outcome further supports NWU as a biologically meaningful oedema marker, linking lower ΔNWU after EVT to a reduced risk of malignant swelling.

Additionally, our results have implications for the biological characterisation of tissue injury in EVT-treated large-core stroke. Specifically, quantitative oedema metrics such as NWU may complement extent-based imaging markers by capturing ischaemic water uptake not conveyed by ASPECTS or core volume alone. However, the present study was not designed to establish a treatment-selection algorithm, and our data do not support the routine use of NWU at present to select or deselect patients for EVT in daily clinical practice. In particular, no validated treatment threshold can be derived from this analysis to justify withholding EVT in otherwise eligible patients. Thus, based on the currently available randomised evidence, treatment of otherwise eligible patients with large-core/low-ASPECTS stroke remains justified.^40^

At present, NWU should therefore be regarded primarily as a quantitative biomarker for biological stratification and future research rather than as an established gatekeeping tool for treatment allocation. Broader clinical translation will require prospective validation and fully automated, standardised NWU pipelines.^6^

This study has limitations. The analysis was based on a secondary as-treated cohort, which may introduce selection bias. Although NWU quantification has been validated in prior work, it currently relies on dedicated post-processing. NWU measurements performed on NCCT can be influenced by timing, enhancement heterogeneity and residual iodine after EVT. Of note, follow-up imaging was performed after a mean time from admission of 25 h, when residual contrast leakage is expected to be markedly reduced compared with immediate post-procedural imaging.^41^ Importantly, the mediation analysis should be interpreted cautiously. Because ΔNWU was measured after treatment, the estimated indirect effects are model-dependent and rely on assumptions that cannot be fully verified, including the absence of unmeasured confounding between the mediator and functional outcome and the absence of mediator–outcome confounders affected by treatment. Although mediation models were adjusted for major prespecified baseline covariates, post-randomisation factors such as reperfusion status, complications, blood pressure management, ICU-based measures, decompressive surgery, withdrawal or limitation of care and other aspects of subsequent clinical management may have influenced functional outcome and may also have been related to oedema progression. The routine follow-up CT at approximately 24 h temporally preceded at least part of these downstream decisions, which may reduce but does not eliminate the risk of post-treatment confounding. Accordingly, the mediation findings should be regarded as hypothesis-generating and mechanistically informative rather than as a precise confounder-free causal decomposition of the EVT effect. In addition, malignant oedema was recorded according to the trial imaging protocol as a visual, categorical marker of space-occupying infarction and was not intended as a detailed morphometric oedema metric. For this reason, the primary oedema marker of the present analysis was NWU, which provides a more quantitative CT-based measure of ischaemic water uptake. Fully automated NWU pipelines (registration, segmentation, mirrored sampling, artefact handling) will be needed to minimise reader input and enable prospective deployment and external validation. Further prospective studies, ideally incorporating dual-energy CT and MRI-based oedema measures, are warranted to corroborate and extend our findings.^41–43^ Accordingly, the present findings should not be interpreted as supporting immediate routine NWU-based treatment selection in clinical practice. In addition, the reported proportions mediated, including the estimates of 34% for ΔNWU and 14% for ΔASPECTS, should be interpreted as approximate, model-derived indicators of relative pathway strength rather than as precise quantitative partitions of the EVT treatment effect.

In conclusion, this study suggests that oedema reduction represents an important model-supported pathway associated with the treatment effect of EVT in patients with large infarct lesions. These findings highlight the importance of considering not only penumbra salvage but also secondary tissue pathophysiology when interpreting treatment effects in extensive ischaemic injury. Quantitative oedema imaging biomarkers such as NWU may help refine biological stratification in future studies, but their role in routine treatment selection requires prospective validation and automated implementation.

## Supplementary Material

SUPPLEMENT_aakag055
