# Supplementary material for: Oedema reduction mediates thrombectomy benefit in large core stroke: secondary analysis of the TENSION trial
Source: Eur Stroke J. 2026 Jun 4;11(6):aakag055. doi: 10.1093/esj/aakag055 (PMC13235715; doi:10.1093/esj/aakag055)
Supplement: SUPPLEMENT_aakag055 [file supplement_aakag055.docx]

**Edema Reduction Mediates Thrombectomy Benefit in Large Core Stroke: Secondary Analysis of the TENSION Trial**

SUPPLEMENT

**Methods**

The trial enrolled patients with acute ischemic stroke due to large vessel occlusion in the anterior circulation and established large infarcts (ASPECTS 3–5) within 11 hours of symptom onset. Patients were randomized to receive endovascular thrombectomy (EVT) plus best medical treatment (BMT) or BMT alone.

Inclusion criteria: age of 18 years or older, pre-stroke independence defined as mRS score of 0-2, NIHSS score of <26, acute ischemic stroke due to occlusion in the M1 segment of the middle cerebral artery or the intracranial segment of the distal internal carotid artery (ICA), established large infarct on admission defined as an ASPECTS of 3-5 as assessed locally and randomization within 11h of symptom onset or last known to be well, with expected completion of EVT within 12h.

Mediation model: We applied a mediation framework as described by Zhao, Lynch, and Chen, using changes in net water uptake (ΔNWU), ASPECTS (ΔASPECTS), and, exploratorily, malignant edema (MMI) status as mediator variables. Age, baseline NIHSS, baseline ASPECTS, time from symptom onset to imaging, and tFU were included as covariates, as appropriate. For mediation analysis, three relationships were assessed: (1) the total effect of EVT on the outcome; (2) the effect of EVT on each mediator; and (3) the association between each mediator and the outcome. After confirming the statistical significance of these associations, indirect (mediated) and direct effects were estimated. To meet model assumptions regarding normality and homoscedasticity of residuals, ΔNWU and ΔASPECTS were log-transformed (log+1). Indirect effects were additionally examined using nonparametric bootstrap with 5,000 resamples as a sensitivity analysis. Bootstrap confidence intervals were used to assess the robustness of the mediation findings. The proportion of the total effect mediated by each variable was then calculated. Agreement between CTP-based versus NCCT-based NWU calculation was assessed using within-subject differences (paired t-test), intraclass correlation coefficients (ICC), and Bland–Altman analysis.

**Supplemental Figure 1: Mediation models**

**
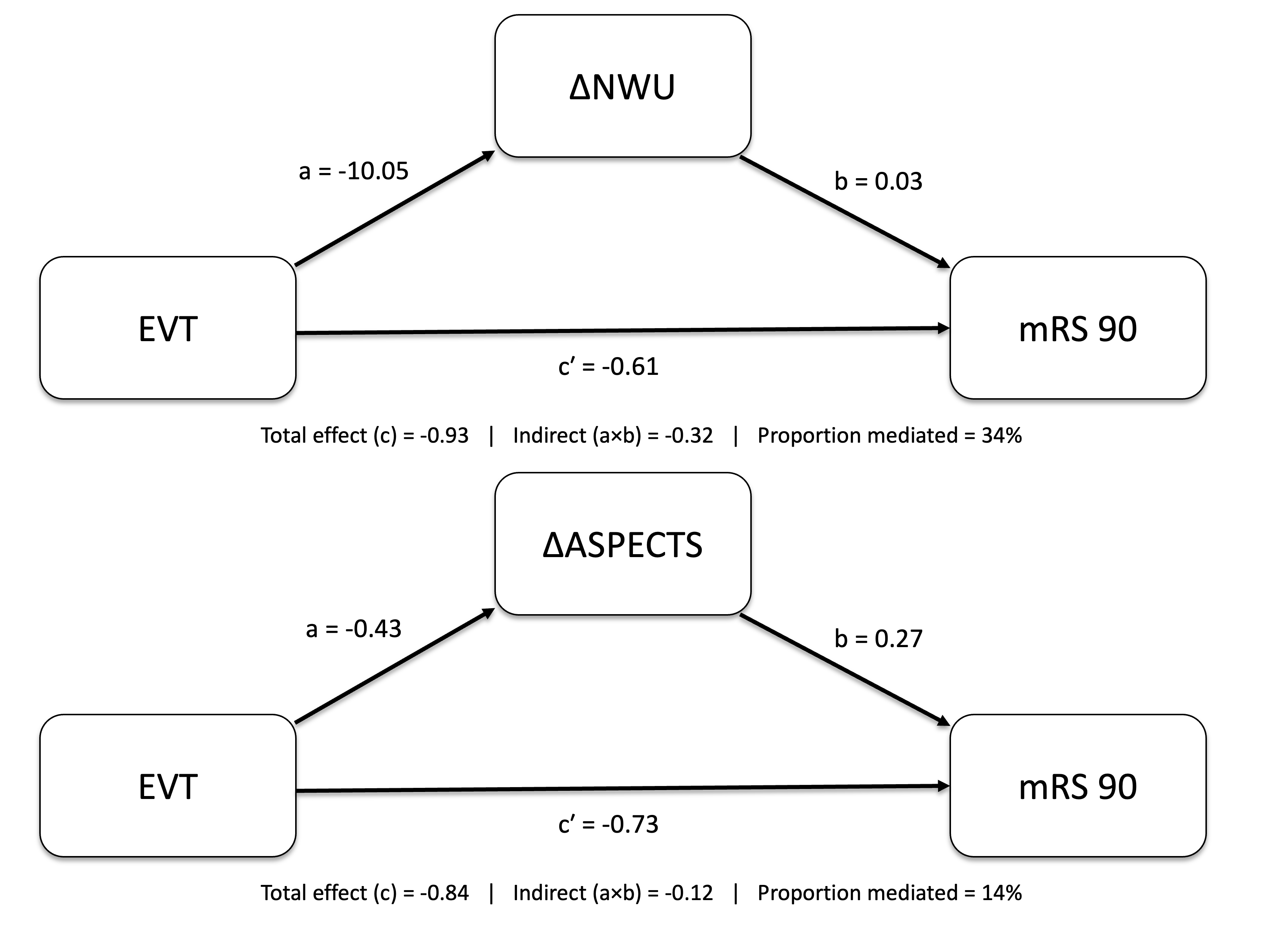
**

Model A (EVT → ΔNWU → modified Rankin Scale at 90 days): EVT was associated with lower edema progression (path a), and larger ΔNWU was associated with worse mRS at day 90 (path b). In the primary mediation model, 34% of the overall EVT effect was associated with ΔNWU. Sensitivity analyses using nonparametric bootstrap with 5,000 resamples yielded directionally similar results. Model B (EVT → ΔASPECTS → mRS): 14% of the EVT effect was associated with ΔASPECTS in the primary model, whereas bootstrap-based sensitivity analyses suggested a weaker and less robust mediation signal. Models were adjusted for age, baseline NIHSS, baseline ASPECTS, and onset-to-imaging time; in ΔNWU-based analyses, time from admission to follow-up CT was additionally included.

Abbreviations: mRS, modified-Rankin-Scale; EVT, endovascular thrombectomy; BMT, best medical treatment; NWU: net water uptake

**Supplemental Table 1.** Path–Coefficient Mediation Analysis (ΔNWU)

| **Path** | **Coefficient** | **95 % CI** | **p-value** |
| --- | --- | --- | --- |
| a (EVT → ΔNWU) | -10.05 | −13.0 - −7.1 | <0.01 |
| b (Δ‑NWU → mRS 90) | +0.03 | +0.02 - +0.05 | <0.01 |
| c (Total effect) | -0.93 | −1.30 - −0.55 | <0.01 |
| c′ (Direct effect) | -0.61 | −0.95 - −0.28 | 0.01 |
| Indirect (a×b) | -0.32 | −0.54 - −0.09 | <0.01 |
| Proportion mediated | 0.34 | 0.18 - 0.50 | — |
|  |  |  |  |

Abbreviations: EVT, endovascular thrombectomy; ΔNWU, change in net water uptake; mRS, modified Rankin Scale; CI, confidence interval; a, effect of EVT on ΔNWU; b, effect of ΔNWU on mRS at 90 days; c, total effect of EVT on mRS; c′, direct (non-mediated) effect; Indirect (a×b), mediated effect; Proportion mediated, fraction of the total effect (c) explained by the indirect path.

**Results**

***Subanalysis: Agreement of CTP- and CTA-assisted core ROIs for NWU***

In a predefined subset of participants with admission CTP available, NWU values derived from baseline NCCT were compared using two alternative core ROI definitions: (1) CTP-CBV-core derived ROIs co-registered to NCCT (core defined on parametric CBV as in Minnerup et al), and (2) CTA-SI derived ROIs co-registered to NCCT as described above.

In this subset with admission CTP available (n = 28), NWU on baseline NCCT showed high agreement between CTP-CBV– and CTA-SI–derived ROI definitions. The mean within-subject difference (CTP-based NWU minus CTA-assisted NWU) was 0.7% (SD 3.6%), which was not different from 0 (paired t-test p = 0.18). The ICC was 0.91 (95% CI 0.82–0.96). Bland–Altman analysis demonstrated a mean bias of −0.02%, with 1/28 cases outside the 95% limits of agreement.

**Acknowledgments**

We thank the patients and their families for participating in the TENSION trial, the members of the data and safety monitoring board, the Koordinierungszentrum Klinische Studien Heidelberg, the European Society of Minimally Invasive Neurological Therapy (ESMINT), the International Consortium for Health Outcomes Measurement (ICHOM), and the Stroke Alliance for Europe (SAFE) for their collaboration on the TENSION project. Participating centers and site investigators of the TENSION trial are listed in the supplemental material.

***Funding***

EU Horizon 2020 research and innovation programme (754640).

***Data sharing statement***

Data that underlie the results reported in this article, after de-identification, will be made available on request beginning 12 months and ending 36 months following publication of the TENSION trial to investigators whose proposed use of the data has been approved by the TENSION steering committee.(46)

***Disclosures***

**G Broocks** reports compensation from Eppdata GmbH for consultant services and a research grant from the Foundation of the American Society of Neuroradiology.

**L Meyer** reports compensation from Eppdata GmbH for consultant services.

**H Kniep** reports compensation as speaker from Asklepios Kliniken, travel funding from Penumbra, Inc., an ownership stake in Eppdata GmbH and compensation from Eppdata GmbH for consultant services.

**G Thomalla** reports funding from the European Commission (EUHorizon 2020 research and innovation programme, 754640; payments to the institution); personal consulting fees from Acandis, AstraZeneca, Bayer, Boehringer Ingelheim, and Stryker; personal payment or honoraria for lectures, presentations, speakers bureaus, manuscript writing, or educational events from Acandis, Alexion, Marin, Bayer, Boehringer Ingelheim, BristolMyersSquibb/Pfizer, Daiichi Sankyo, and Stryker; participation as DSMB member for the TEA Stroke Trial (no payments) and ReSCInD trial (no payments); work as a speaker of the Commission for Cerebrovascular Diseases of the German Society of Neurology (DGN; no payments); and membership of the Board of Directors of the European Stroke Organisation (ESO; no payments).

**M Bendszus** reports funding from EU Horizon 2020 and Deutsche Forschungsgemeinschaft (payments to the institution); honoraria for lectures from Novartis, Boehringer Ingelheim, and Seagen; and consulting fees from NeuroScios and Boehringer Ingelheim and is an editor in chief of Clinical Neuroradiology (Springer).

**AH Aamodt** reports unrestricted research grants from Boehringer Ingelheim; honoraria for lectures from BMS/ Pfizer, Teva, Roche, Abbvie, Lundbeck, and Novartis; and participation in Advisory Boards for MSD, BMS/Pfizer, Lundbeck, Lilly, and Abbvie.

**B Fuentes** reports research grants from Carlos III Institute of Health; personalpayment for educational lectures from Servicio Madrileño de Salud; payment for lectures from Euromedice to the institution; personal payment for educational lectures from Takeda; support for attending meetings from Daiichi Sankyo; receipt of materials for research from Abbot.

**MD Hill** reports funding from Nil; grants to the University of Calgary for the TEMPO-2 trial from Boehringer Ingelheim, Biogen, NoNO (ESCAPE-NA1 trial and ESCAPE-NEXT trial), Canadian Institute for Health Research (ESCAPE-NA1 trial and ESCAPE-NEXT trial), Medtronic (HERMES collaboration), Alberta Innovates (QuICR Alberta Stroke Program); that some of the funds were used for the ESCAPE-NA1 trial from Alberta Innovates; consulting fees from Sun Pharma Brainsgate (paid work for adjudication of clinical trial outcomes); US patents 62/086,077 (licensed to Circle NVI) and 10,916,346 (licensed to Circle NVI); private stock ownership from Circle and PUreWeb; participation as data and safety monitoring committee chair of the RACECAT trial (end 2020), the Oncovir Hiltonel trial (ongoing), and the DUMAS trial (ongoing); participation as a data and safety monitoring committee member of the ARTESIA trial (ongoing), and the BRAIN-AF trial (ongoing); and is president of the Canadian Neurological Sciences Federation (not for profit) and a Board member of the Canadian Stroke Consortium (not for profit).

**A Krajina** reports grants from the European Commission for the TENSION study (payment to the institution).

**L Pierot** reports consulting fees from Balt, Microvention, and Phenox; and support for attending meetings or travel for the TENSION investigator meeting (transport and accommodation was reimbursed by the organization).

**CZ Simonsen**reports grants from Tryg Foundation and Health Research Foundation of Central Denmark Region.

**J Fiehler** reports funding from the European Commission; personal consulting fees from Acandis, Cerenovus, Medtronic, Microvention, Phenox, Stryker, and Roche; consulting at Philips (no payments); payment or honoraria for lectures, presentations, speakers bureaus, manuscript writing or educational events from Penumbra and Tonbridge; support for attending meetings or travel from Medtronic and Penumbra; stock or stock options from Tegus Medical, Eppdata, and Vastrax; and participation in a Data Safety Monitoring Board or Advisory Board at Phenox (personal fees) and Stryker (personal fees) and is a past president of ESMINT.

**A Wouters** reports consultancy fees from Bayer.

**R Lemmens** has no personal disclosures, but reports institutional fees paid to KU Leuven for consultancy by Boehringer-Ingelheim and iSchemaview
